# Micronization Combined Ultrasound-Assisted Extraction Enhances the Sustainability of Polyphenols from Pineapple and Lemon Peels Utilizing Acidified Ethanol

**DOI:** 10.3390/foods14162872

**Published:** 2025-08-19

**Authors:** Yen-Chieh Lee, Yi-Chan Chiang, Min-Hung Chen, Po-Yuan Chiang

**Affiliations:** 1 Department of Food Science and Biotechnology, National Chung Hsing University, No. 145 Xingda Road, South Dist., Taichung City 40227, Taiwan; 2 Agriculture and Food Agency, Ministry of Agriculture, No. 37, Nanhai Rd., Zhongzheng Dist., Taipei City 100212, Taiwan

**Keywords:** byproduct, micronization, citric acid, ultrasound-assisted extraction, sustainable valorization, contour plot analysis

## Abstract

Pineapple and lemon processing generates large volumes of peel waste, which is a valuable source of dietary polyphenols and flavonoids with potent antioxidant activity. This study employed a strategy of micronization and ultrasound-assisted extraction (UAE) with acidified ethanol to valorize pineapple peel (PP) and lemon peel (LP). Physicochemical characteristics, total polyphenol content, total flavonoid content, and antioxidant activities (DPPH, FRAP, and ABTS^+^) were evaluated under varying particle sizes, ethanol concentrations, extraction times, and pH conditions. Optimal extraction was achieved with 30 min of UAE using 75% ethanol acidified with citric acid at pH 5 for PP (96.6 µm) and pH 4 for LP (91.7 µm). These conditions maximized polyphenol yields, with the LP micropowder produced containing 65.7 µg/mg of hesperidin and 23.2 µg/mg of eriocitrin. Contour plots and principal component analysis confirmed that the antioxidant extraction selectivity of micropowder was dependent on pH and extraction time. Microstructural and Fourier-transform infrared spectroscopy analyzes further supported that short-dried period and a lower particle size significantly improve solute release. This study not only demonstrates the efficacy of micronization and UAE in enhancing the selective extraction of antioxidants from fruit peels but also offers a digital visualization strategy for optimizing extraction processes to support sustainable bioprocessing and functional ingredient development.

## 1. Introduction

Pineapple (*Ananas comosus* (L.) Merr.) and lemon (*Citrus limon* (L.) Brum.) are significant economic crops in Taiwan. As reported by the Food and Agriculture Organization (FAO), their annual production amounts in 2025 were 378,000 and 33,000 metric tons, respectively. Most of the fruit pulp is processed into various food products, including juice, jam, stuffing, dried fruit, and canned goods. However, the accumulation of large quantities of by-products is incompatible with the environmental sustainability objectives established by the United Nations, such as the disposal of peel, pomace, and seeds, with peel accounting for over 30% of the by-products [1]. Peel is mostly used in the production of low-value-added natural antioxidants and intestinal health promoters, such as feed and fertilizer. Consequently, the integration of environmental-friendly extraction techniques to enhance the value of by-products has recently emerged as a significant topic of discussion [2,3,4]. Numerous studies have demonstrated that peel is a rich source of dietary fiber, pectin, and polyphenol compounds, including small-molecule phenolic acids and flavonoids. These compounds have been extensively studied for their potent antioxidant properties and the potential to mitigate the risk of cardiovascular disease and cancer by reducing oxidative stress [5,6,7].

Recent studies have increasingly focused on the valorization of fruit peels, such as pineapple peel (PP) and lemon peel (LP), due to their high polyphenol content. For example, Yahya et al. [8] demonstrated that ultrasound-assisted extraction significantly enhanced the recovery of total phenolic and flavonoid contents from PP, outperforming conventional Soxhlet extraction in both efficiency and antioxidant capacity. Similarly, Durmus and Kilic-Akyilmaz [9] evaluated the phenolic composition of LP extracts (LPEs) obtained via ultrasound-assisted extraction and enzyme-assisted extraction and reported that non-extractable phenolics retained in the peel matrix exhibited considerable antioxidant, antihypertensive, and antidiabetic activities. These findings highlight the potential of environmental-friendly extraction technologies for extracting valuable bioactive compounds from fruit by-products. However, most studies emphasize single-variable optimization or response surface methodology do not comprehensively explore the combined effects of parameters such as particle size, pH, and solvent polarity under non-thermal conditions. Moreover, comparative evaluations of different fruit peels, such as LP and PP, using a standardized extraction framework are scarce.

The stability of antioxidants in the peel is influenced by various external and internal factors. External factors include temperature, solvent concentration, and extraction time; internal factors include particle size and pH value. As reported in Chew et al. [10], increasing the extraction temperature from 25 °C to 65 °C increased total polyphenols and total flavonoids. However, the scavenging activity of DPPH and ABTS^+^ decreased, indicating that thermosensitive polyphenols are degraded at temperatures that exceed the optimal range. Different ethanol concentrations also influence their antioxidant capacity. Previous research has indicated that extraction time is a significant factor affecting antioxidant capacity and total polyphenol content. For instance, the optimal extraction time for ultrasound-assisted polyphenol extraction of PP is 30 min, but the extract degrades after 45 min [11]. The particle size of the solute also influences extraction capacity in an inversely proportional manner. As reported in Chen et al. [12], the total flavonoids increased 28.2-fold after micronization of okara. As reported in Chupin et al. [13], maritime pine bark powder with a diameter of 0.1 mm had the highest extraction efficiency (13.2%), along with a total polyphenol content of 39.5 mg gallic acid equivalent (GAE) (g dry weight (DW))^−1^. Simultaneously, low-acid solvents could mitigate enzymatic oxidation, thereby augmenting total polyphenols and antioxidant capacity (*p* < 0.05). Notably, acidified ethanol (pH 4) exhibited the most pronounced effect in this regard, likely due to the reduced activity of polyphenol oxidase under low pH extraction conditions [14]. Consequently, inappropriate conditions can accelerate the cleavage or degradation of phenols, including gallic acid, ferulic acid, and *p*-coumaric acid, as well as flavonoids such as hesperidin and eriocitrin [5,6].

In recent years, environmental-friendly extraction technology has been advocated as a means of addressing the limitations of traditional extraction methods, such as Soxhlet extraction, maceration, and hydrodistillation. These limitations include extended extraction times, the use of non-environmentally friendly solvents, and low extraction selectivity for thermally sensitive components [15]. In response to the growing emphasis on environmental sustainability, non-toxic or food-grade solvents are increasingly being employed to replace traditionally used organic solvents, such as hexane, benzene, chloroform, petroleum ether, and acetone [16]. Although supercritical fluid technology eliminates the disadvantage of solvent residue, its high input cost and energy consumption restrict its widespread application [17,18]. Ultrasonic extraction technology, on the other hand, is characterized by its rapidity, efficiency, and ability to effectively reduce solvent usage under highly reproducible conditions, which is attributed to its high unit extraction efficiency [19]. Furthermore, non-thermal extraction conditions are versatile and can be employed in the extraction of natural active ingredients, including the synergistic additive effect of the cavitation effect and the sponge effect. The cavitation effect generates numerous minute bubbles between the solvent and the solute, resulting in directed micro-jetting that effectively destroys the cell wall. Concurrently, as the cavitation bubble undergoes continuous transformation under the contraction pressure, minute vortices (microturbulences) are generated around the cell wall, leading to the collapse of the bubble and the generation of mechanical shear force [19]. The sponge effect results in the formation of numerous microchannels on the cell wall and cytoplasm, facilitating the release of functional components within the cell [20,21,22]. Numerous studies have investigated extraction techniques for fruit peel polyphenols. However, prior research frequently concentrated on extraction parameters or thermal methods, lacking a comprehensive integration of multiple influential factors, including particle size, solvent polarity, and extraction pH under non-thermal conditions. Furthermore, there is a lack of systematic comparative studies that specifically assess pineapple and lemon peels within a coherent extraction framework. The present study sought to address this gap by employing a novel approach that integrates micronization and ultrasound-assisted extraction (UAE) using acidified ethanol on pineapple and lemon peels. Furthermore, this study presents data-driven optimization through the use of contour plots and principal component analysis (PCA), providing clear visual guidelines for extraction conditions. These insights facilitate scalable and environmentally sustainable processing strategies, improving the sustainability and functional use of fruit peel by-products.

## 2. Materials and Methods

### 2.1. Preparation of Pineapple and Lemon Peel Micropowder

Pineapple and lemon peels were procured from the same vendor (Taichung City, Taiwan) and subjected to freeze-drying (−40 °C, 48 h) and hot-air drying (55 °C, 48 h) before milling and sieving. Frozen peel slices were freeze-dried for 48 h in a laboratory freeze-dryer (FD4.5-8P-L, Kingmech Co., Ltd., New Taipei City, Taiwan). The condenser was maintained at −40 °C, the chamber pressure at 0.05 ± 0.01 mbar, and the shelf temperature at −20 °C for the first 42 h (primary drying) before ramping to 20 °C for the final 6 h (secondary drying). A K-type thermocouple inserted into the center of a peel slice confirmed a product temperature of −18 ± 1 °C during primary drying, increasing to 20 °C during secondary drying. The forced-convection oven with airflow of hot-air drying was at 0.26 m^3^ min^−1^ ≈ 18 ACH (air changes per hour), and superficial velocity set as 1.0 m s^−1^. Subsequently, the dried peel was crushed by a small grinder (RT-08, Rong Tsong Precision Co., Ltd., Taichung City, Taiwan) and sieved (through 60, 80, and 100 mesh of stainless-steel sieves) to produce PP powder and LP powder (the water content of PP powder and LP powder are 6.91 and 5.63 g_water_/g_DW_), which were then vacuum-packed under light-protected conditions, pre-stored at −18 °C, and subsequently transferred to −80 °C for storage until further use.

### 2.2. Chemicals

All chemicals used were of analytical grade from Sigma-Aldrich Co. (St. Louis, MO, USA). All solutions were prepared using deionized water with a resistivity of no < 18.2 MΩ cm^−1^.

### 2.3. Screening of Extraction Conditions for Micropowder

Ultrasonic extraction, using an ultrasonic vibrator (Series Lu101, Ringtech Instruments Co., Ltd., Taichung, Taiwan) and different solvents, was performed to extract the PP and LP micropowder at 10 ± 5 °C. The applied mass of peel powder and the used volume of solvent were 1:10 (*w*_sample_/*v*_solution_). The pH was subsequently adjusted by citric acid to optimize the extraction conditions for further antioxidant characterization. Additionally, extraction efficiency was compared between ultrasonic extraction and a conventional method of stirring at 250 rpm for 30 min (Table 1).

### 2.4. Physical Properties of Micropowder

#### 2.4.1. Imaging of Appearance

Surface images were captured using an optical dissecting microscope (Model SMZ800, Nikon Co., Tokyo, Japan) in conjunction with a digital SLR camera (Model 450D, Canon Co., Tokyo, Japan). Subsequently, the microstructure was captured with a field emission scanning electron microscope (Model JSM-7800F Prime, JEOL Ltd., Tokyo, Japan). The specimen was positioned on an aluminum rod with a platinum layer coated with platinum-palladium (Model JEC-3000FC, JEOL Ltd.) and then examined at an accelerating voltage of 3 kV with operational parameters identical to those of a previous study [23].

#### 2.4.2. Color Analysis

Adhering to the methodology outlined in a previous study [24], color was measured with a colorimeter (Model ZE2000, Nippon Denshoku Industries Co., Tokyo, Japan). Calibration was performed using a whiteboard (X = 92.81, Y = 94.83, and Z = 111.71) and a blackboard (X = 3.26, Y = 3.00, and Z = 2.29).

### 2.5. Determination of Physicochemical Properties of Micropowder

One gram of dry micropowder was combined with 10 mL of deionized water and subjected to ultrasonic extraction for 30 min using an ultrasonic vibrator (Series Lu101, Ringtech Instruments Co., Ltd.). The solutions were centrifuged at 3820× *g* for 20 min at ambient temperature, and the supernatant was collected and passed through a 0.22 μm polytetrafluoroethylene filter (Waters Co., Milford, MA, USA). The extract was stored at −20 °C until it could be analyzed later.

#### 2.5.1. pH and Total Soluble Solids

The pH of extracts obtained previously (Section 2.4) was measured using an electrode (JQ-EP-122, Suntex Instruments Co., Ltd., New Taipei, Taiwan) and a pH meter (Model SP-2300, Suntex Instruments Co., Ltd., New Taipei, Taiwan) after calibration. Total soluble solids (TSS) were determined using a refractometer (Model MASTER-M, Atago Co., Tokyo, Japan) at ambient temperature (25 °C) [24].

#### 2.5.2. Water-Holding Capacity (WHC) and Oil-Holding Capacity (OHC)

All micropowder was thoroughly mixed with deionized water and sunflower oil at a ratio of 1:10 (*w*/*v*) and 1:10 (*w*/*w*), respectively. To determine water-holding capacity (WHC) and oil-holding capacity (OHC), the micropowder was immersed in deionized water and non-blended sunflower oil for 24 h and 30 min, respectively. These solutions were then centrifuged at 1300× *g* for 20 min at ambient temperature. The supernatant was collected and passed through a 0.22 μm polytetrafluoroethylene filter [25]. The WHC and OHC values were calculated using Equations (1) and (2).

WHC (g/g) = (V_Hb_ − V_Ha_) × 1.00/W_a_ (1)

OHC (g/g) = (V_Ob_ − V_Oa_) × 0.85/W_a_ (2)

where V_Hb_, V_Ha_, V_Ob_, V_Oa_, and W_a_ denote the volume of water before and after sample addition, the volume of oil before and after sample addition, and the weight of the sample, respectively.

#### 2.5.3. Particle Size

The particle size distribution was determined using a laser particle size analyzer (Bettersizer S, Model BT-801 ACD System, Dandong Bettersize Instruments Ltd., Dandong, China). Deionized water was used as the dispersing phase. The average particle size was expressed as D_50_ [26].

#### 2.5.4. Fourier-Transform Infrared Spectroscopy Analysis

All samples were measured using a Fourier-transform infrared spectrometer (Model Nicolet 6700, Thermo Fisher Scientific Co., Waltham, MA, USA) in conjunction with a mercury cadmium telluride detector. Spectra were recorded over the range of 500–4000 cm^−1^, with a resolution of 2 cm^−1^ and a scan time of 30 s [6].

### 2.6. Antioxidant Characterization Evaluation

#### 2.6.1. Total Polyphenols

We mixed 70 μL of each of the extracts from Section 2.3 with an equal volume of Folin–Ciocalteu reagent (Sigma-Aldrich Co., St. Louis, MO, USA) in a 96-well microplate and allowed the mixture to stand for 3 min. Then, 35 μL of 10% (*w*/*v*) sodium carbonate aqueous solution was added as an alkalizing agent, and the mixture was incubated for 30 min at ambient temperature in dark conditions. The absorbance wavelength was then measured at 735 nm using an ELISA reader (Model SPECTROstar Nano, BMG Labtech Co., Ortenberg, Germany) at ambient temperature in dark conditions. Gallic acid (1000, 500, 250, 125, 62.5, and 31.25 mg kg^−1^) was used as the standard and quantified to mg (100 g DW)^−1^ using calibration curves [27].

#### 2.6.2. Total Flavonoids

We mixed 50 μL of each of the extracts from Section 2.3 with 300 μL of deionized water and 150 μL of 5% (*w*/*v*) sodium nitrite aqueous solution. After the mixture was allowed to stand for 6 min, 125 μL of 2.5% (*w*/*v*) aluminum chloride aqueous was added as a complexing agent, followed by 125 μL of 2% (*w*/*v*) sodium hydroxide aqueous as an alkalizing agent. The mixture was then incubated for 15 min at ambient temperature in dark conditions. The absorbance was measured at 415 nm using an ELISA reader (Model SPECTROstar Nano, BMG Labtech Co., Ortenberg, Germany) at ambient temperature in dark conditions. Quercetin (1000, 500, 250, 125, 62.5, and 31.25 mg kg^−1^) was used as the standard and quantified to mg (100 g DW)^−1^ using calibration curves [28].

#### 2.6.3. 2,2-Diphenyl-1-picrylhydrazyl Radical-Scavenging Activity

We mixed 10 μL of each of the extracts from Section 2.3 with 75 μL of 0.5 mM 2,2-diphenyl-1-picrylhydrazyl (DPPH) solution (in methanol) and 40 μL of 100 mM Tris-HCl buffer (pH 7.4). The mixture was incubated for 30 min at ambient temperature in dark conditions. The absorbance was measured at 517 nm using an ELISA reader (Model SPECTROstar Nano, BMG Labtech Co., Ortenberg, Germany) at ambient temperature in dark conditions. Trolox (1000, 500, 250, 125, 62.5, and 31.25 mg kg^−1^) was used as the standard and quantified to mg (100 g DW)^−1^ using calibration curves [24].

#### 2.6.4. Ferric Ion Reducing Power

Ferric ion reducing antioxidant power (FRAP) reagent was prepared in a 300 mM acetate buffer (pH 3.6) containing 10 mM 2,4,6-tripyridyl-s-triazine (TPTZ) (in 40 mM HCl) as a chelating agent chromogen and 20 mM ferric chloride as an oxidant at a ratio of 10:1:1 (*v*/*v*/*v*). Then, 20 μL of the extracts from Section 2.3 was reacted with 150 μL of FRAP reagent at 37 °C for 10 min in dark conditions. The absorbance was measured at 593 nm using an ELISA reader (Model SPECTROstar Nano, BMG Labtech Co., Ortenberg, Germany). Trolox (1000, 500, 250, 125, 62.5, and 31.25 mg kg^−1^) was used as the standard and quantified to mg (100 g DW)^−1^ using calibration curves [28].

#### 2.6.5. 2,2′-Azino-bis(3-ethylbenzothiazoline-6-sulphonic Acid) Free Radical Scavenging

2,2′-azino-bis(3-ethylbenzothiazoline-6-sulphonic acid) (ABTS^+^) reagent was prepared by mixing ABTS^+^ (7 mM) and potassium persulfate (2.45 mM) at a ratio of 2:1 (*v*/*v*), and allowing the mixture to incubate in darkness for 12 h. This stock solution was diluted with 100 mM Tris-HCl buffer (pH 7.4) to achieve an absorbance of 0.70 ± 0.02 at 734 nm.

Then, 50 μL of each of the extracts from Section 2.3 was mixed with 1200 μL of ABTS^+^ reagent and allowed the mixture to react for 30 min at ambient temperature in dark conditions. The absorbance was measured at 734 nm using an ELISA reader (Model SPECTROstar Nano, BMG Labtech Co., Ortenberg, Germany) at ambient temperature in dark conditions. Trolox (1000, 500, 250, 125, 62.5, and 31.25 mg kg^−1^) was used as the standard and quantified to mg (100 g DW)^−1^ using calibration curves [29].

### 2.7. Functional Component Assessment of Pineapple Peel (PPE) and Lemon Peel (LPE)

#### 2.7.1. Phenolic Acid Analysis

All extracts from Section 2.3 were filtered through a 0.22-μm PTFE membrane (Waters Co., Milford, MA, USA) to remove impurities and then analyzed using high-performance liquid chromatography with UV detection (HPLC-UV), which consisted of an autosampler (Model PN5300, Postnova Co., Salt Lake City, UT, USA), a chromatographic pump (Model Chromaster 5110, Hitachi Co., Tokyo, Japan), a column (Mightysil RP-18GP, 250 mm × 4.6 mm i.d., 5.0 μm; Kanto Co., Tokyo, Japan), and a UV-VIS detector (Model Chromaster 5420, Hitachi Co., Tokyo, Japan).

The mobile phase, composed of A (methanol:acetic acid:deionized water = 10:2:88, *v*/*v*/*v*) and B (methanol:acetic acid:deionized water = 90:2:8, *v*/*v*/*v*), was used for gradient elution. The elution gradient consisted of the following phases: 0–15 min at 85% A (15% B), 15–20 min at 50% A (50% B), 20–35 min at 30% A (70% B), and 35–60 min at 100% A. The injection volume was 15 μL, and the flow rate was 0.8 mL/min at ambient temperature (25 °C). The absorbance was measured at 280 nm. Qualitative and quantitative analyses were performed based on different retention times of gallic acid, caffeic acid, *p*-coumaric acid, ferulic acid, and cinnamic acid, calculated using calibration curves established from external standards. Calibration curves were established using five concentrations from 3.125 to 100.000 µg (mg DW)^−1^ for gallic acid, caffeic acid, and *p*-coumaric acid. Ferulic acid was assessed at five concentrations, ranging from 0.391 to 25 µg (mg DW)^−1^, with correlation coefficients (R^2^) exceeding 0.994 in all instances (Figure S2) [30].

#### 2.7.2. Polyphenols Analysis

All extracts from Section 2.3 were analyzed using the same HPLC-UV system as described in Section 2.7.1. The mobile phase, composed of A (0.1% (*w*/*v*) formic acid aqueous solution) and B (acetonitrile), was employed for gradient elution. The elution gradient consisted of the following phases: 0–5 min at 50% A, 5–20 min at 63% A, 20–25 min at 50% A, 25–30 min at 20% A, 30–35 min at 0% A, 35–40 min at 63% A, and 40–45 min at 95% A. The injection volume was 10 μL, and the flow rate was 0.8 mL/min at ambient temperature (25 ± 5 °C). The absorbance was measured at 280 nm. Qualitative and quantitative analyses were performed based on different retention times of hesperidin and eriocitrin, calculated using calibration curves established from external standards. The calibration curves were established using five concentrations from 3.125 to 100.000 µg (mg DW)^−1^ for hesperidin and eriocitrin, with correlation coefficients (R^2^) surpassing 0.987 in all instances (Figure S3) [24].

### 2.8. Contour Plot Visualization of Polyphenolic Compounds and Antioxidant Activities

Contour plots were generated using OriginPro software (ver. 2024, OriginLab Corp., MA, USA) to visually assess the interactions between extraction time (10 to 50 min) and extraction pH (1 to 7) on total polyphenols, total flavonoids, and antioxidant activities, including DPPH radical scavenging, FRAP, and ABTS^+^ assays. All experimental data collected were fitted using second-order polynomial regression equations (Equation (3)). The significance of the fitted model was evaluated using analysis of variance (ANOVA) to ensure accuracy and reliability. Then, the validated regression equations were employed to construct contour plots, providing a clear visualization of optimal extraction conditions based on the intensity and spatial distribution of bioactive compounds and antioxidant responses. This facilitated the interpretation of interactions between and trends in experimental outcomes [31].

(3) $$Y=\beta_{0}+\sum_{i=1}^{k}\beta_{i}X_{i}+\sum_{i=1}^{k}\beta_{ii}X_{i}^{2}+\sum_{i<j}\;\sum_{j=2}^{k}\beta_{ij}X_{i}X_{j}+\varepsilon$$

where *Y* denotes the predicted response (total polyphenols, total flavonoids, or antioxidant activities), *β*_0_ denotes the constant coefficient. *β_i_*, *β_ii_*, and *β_ij_* denote linear, quadratic, and interaction coefficients, respectively. *X_i_* and *X_j_* denote independent variables (extraction time and pH), and *ε* denotes the residual error term.

### 2.9. Statistical Analysis

All results are shown as mean ± standard deviation (*n* = 3). Data were analyzed using one-way analysis of variation. SPSS (ver. 19.0, IBM Co., Armonk, NY, USA) was used for the analysis. Means were compared using Duncan’s multiple range test, with *p* < 0.05 as the threshold for significance. Principal component analysis (PCA) and agglomerative hierarchical clustering (AHC) were performed using XLSTAT software (XLSTAT 2023.3.0, Addinsoft Co., New York, NY, USA).

## 3. Results and Discussion

### 3.1. Appearance of PP and LP

**Figure 1:**
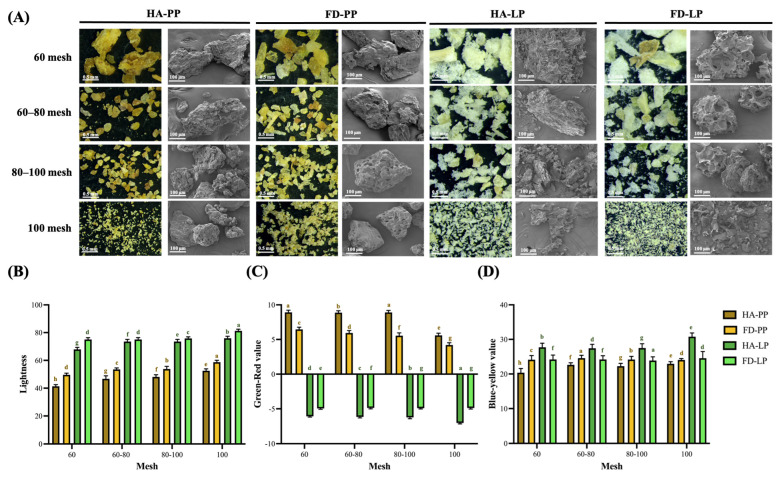
Appearance of pineapple peel and lemon peel powder. (**A**) Microstructure and scanning electron microscopy image, (**B**) Lightness, (**C**) Green–red value, (**D**) Blue–yellow value. Data are presented as means ± standard deviations (*n* = 3). Different letters (a–h) indicate statistically significant differences within the same column (*p* < 0.05). PP, LP, HA, and FD denote pineapple peel powder, lemon peel powder, hot-air drying, and freeze-drying, respectively.

The physical appearance of the peel samples varied notably depending on the drying method employed [32]. As illustrated in Figure 1A, particle morphology changed with different mesh sizes, showing a decrease in particle size with increasing mesh size. Compared to freeze-dried PP powder, freeze-dried LP powder exhibited a broader distribution of particles. This phenomenon is attributable to the structural disintegration induced by freeze-drying of LP, resulting in a brittle and easily fractured matrix [32]. The observed microstructural alterations were closely associated with the drying method. Scanning electron microscopy (SEM) showed surface shrinkage in hot-air-dried samples and numerous porous structures in freeze-dried samples. This aligns with previous studies indicating that hot-air drying causes surface water to evaporate rapidly, leading to case-hardening and subsequent shrinkage [33]. In contrast, freeze-drying effectively removes internal moisture, facilitating the formation of sponge-like, porous three-dimensional networks in both PP and LP [32].

Color changes induced by the drying method were further evaluated (Figure 1B). The lightness values (L*) of PP ranged from 58.7 to 41.4 and those of LP from 81.3 to 67.9, indicating that freeze-drying significantly enhanced brightness compared to hot-air drying. This improvement is likely due to reduced browning from the Maillard reaction under low-temperature conditions [34]. Additionally, smaller particle sizes increase surface area exposure to light, contributing to higher brightness [13,35]. As shown in Figure 1C and Figure 1D, the a* (green–red) values of PP and LP ranged from 8.9 to 4.2 and −6.2 to −4.9, respectively, while the b* (blue–yellow) values ranged from 24.6 to 20.4 and 30.8 to 23.9, respectively. Unlike previous studies, in this study, freeze-drying did not preserve the original color hues. Hot-air drying led to higher redness in PP and higher greenness in LP. Freeze-drying led to an increase in yellowness of PP and a decrease in yellowness of LP, potentially due to increased light reflectance caused by surface porosity [36].

### 3.2. Physicochemical Properties of PPP and LPP

**Figure 2:**
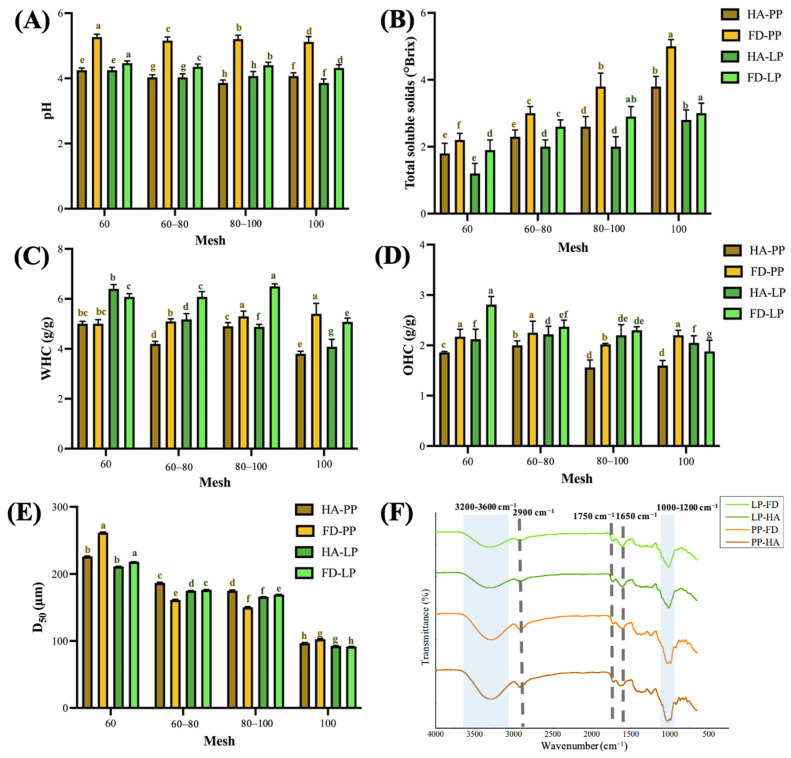
Physicochemical properties of pineapple peel powder and lemon peel powder. (**A**) pH, (**B**) Total soluble solids, (**C**) Water-holding capacity, (**D**) Oil-holding capacity, (**E**) Average particle size, (**F**) Fourier-Transform infrared spectroscope spectrum. Data are presented as means ± standard deviations (*n* = 3). Different letters (a–h) indicate statistically significant differences in the same group (*p* < 0.05). PP, LP, HA, and FD denote pineapple peel powder, lemon peel powder, hot-air drying, and freeze-drying, respectively. The sample measured by FTIR was filtered with a 100-mesh sieve.

The physicochemical properties of powdered plant matrices play a crucial role in the retention and extractability of bioactive compounds with antioxidant potential. Therefore, this section evaluates key physicochemical characteristics of PP and LP (Figure 2). The pH of PP and LP ranged from 5.3 to 3.9 and 4.5 to 3.9, respectively. Hot-air-dried samples exhibited significantly lower pH values (*p* < 0.05), likely due to the dual effect of matrix concentration and the thermal oxidation of acid precursor compounds such as alcohols, aldehydes, and ketones. (Figure 2A) [37]. The TSS of PP were higher than those of LP, ranging from 3.8 to 1.8 °Brix and 3.0 to 1.2 °Brix, respectively. Freeze-dried samples had higher TSS than did hot-air-dried samples (*p* < 0.05), consistent with previous findings that the Maillard reaction–induced conjugation between sugars and proteins during heating reduces TSS content (Figure 2B) [38].

The WHC and OHC of PP were 5.4–3.8 g water (g DW)^−1^ and 2.3–1.6 g oil (g DW)^−1^, respectively, and those of LP were 6.5–4.1 g water (g DW)^−1^ and 2.8–1.9 g oil (g DW)^−1^, respectively. These results confirm the superior WHC and OHC performance of freeze-dried samples (Figure 2C,D). Previous studies indicated that hydrophilic and hydrophobic functional groups, as well as drying methods, influence WHC and OHC. Heat treatment often impairs these capacities [39,40]. The enhanced hydration and oil-retention properties observed in freeze-dried samples may also be attributable to the porous microstructures generated by freeze-drying, as confirmed in SEM images (Figure 1A).

The D_50_ particle sizes of PP and LP ranged from 261.0 to 96.6 µm and 218.2 to 91.7 µm, respectively, with 100-mesh powders corresponding to approximately 96.6 µm and 91.7 µm. Hot-air-dried PP samples exhibited larger D_50_ values than freeze-dried PP samples, possibly due to inefficient drying and case-hardening effects of hot-air treatments, which trap residual moisture and result in coarser particles during grinding (Figure 2E; S4) [41].

Fourier-transform infrared spectroscopy (FTIR) spectra revealed characteristic broad O–H stretching vibrations at 3000–3500 cm^−1^ and C–H stretching at 2900 cm^−1^, likely corresponding to water and cellulose. Notably, the O–H peak intensity was lower in freeze-dried samples, suggesting more effective moisture removal by freeze-drying compared to hot-air drying [42,43]. A distinct peak near 1750 cm^−1^ was attributed to acetyl and ester groups, potentially derived from hemicellulose or from phenolic acids such as ferulic and *p*-coumaric acids that are esterified to polysaccharides in the plant cell wall [44]. The peak intensity of hot-air-dried PP was slightly higher than that of freeze-dried PP, indicating that hot-air-dried PP may contain a higher ferulic acid and *p*-coumaric acid content. The band at 1650 cm^−1^ was associated with O–H bending, indicating the presence of bound water [45]. The absorption band of the C–O–C structure was observed in the 1130–1150 cm^−1^ region, and the peak intensity of LP was greater than that of PP. This region may indicate the ether bond stretching vibration of polysaccharides or glycoside structures, which is speculated to be related to the high flavonoid glycoside content in LP. According to Ernawita et al. [46], LP is rich in a variety of flavonoid glycosides and polymethoxyflavones, indicating that this type of structure may partly contribute to the absorption in this band. Peaks in the 1000–1200 cm^−1^ region represented the fingerprint region of cellulose, confirming the cellulose-rich nature of both fruit peels (Figure 2F) [13,47].

### 3.3. Antioxidant Activities Under Different Extraction Parameters

**Figure 3:**
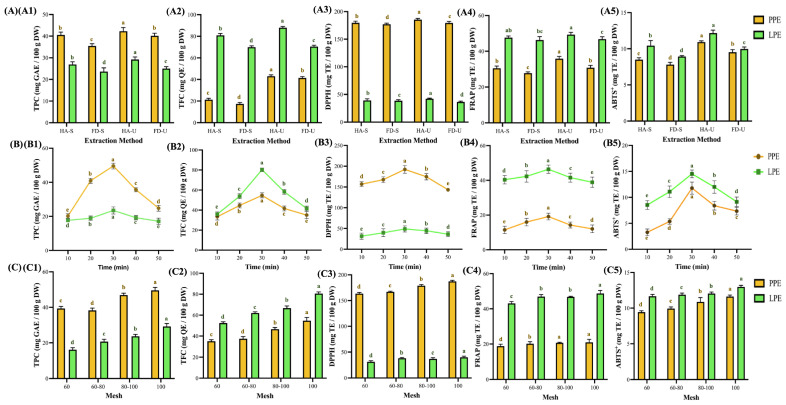
Analysis of functionalities and antioxidant activities of pineapple and lemon peel extracts under different extraction methods (**A**), time (**B**), and mesh (**C**). Peel micropowder was filtered by a 100-mesh sieve, with rows (**B**,**C**) extracted using additional ultrasound-assisted extraction. (1) total polyphenols, (2) total flavonoids, (3) 2,2-diphenyl-1-picrylhydrazyl radical scavenging, (4) ferric ion reducing antioxidant power, and (5) 2,2′-azino-bis(3-ethylbenzothiazoline-6-sulfonic acid) radical scavenging. Data are presented as means ± standard deviations. (*n* = 3). Different letters (a–e) indicate statistically significant differences in the same column (*p* < 0.05). Abbreviation description: PPE and LPE denote pineapple and lemon peel extracts, respectively. HA, FD, -U, -S, denotes the samples used for hot-air drying, freeze-drying, ultrasound-assisted extraction, and magnetically stirred extraction, respectively.

Extraction efficiency is greatly influenced by multiple parameters, including drying method, solvent type, extraction temperature, and duration. Therefore, optimization of these conditions is critical. Figure 3 summarizes the effects of these variables on antioxidant extraction. In Figure 3A, the influence of drying and extraction methods is compared. The total polyphenol content (TPC) of hot-air-dried samples ranged from 42.3 to 26.9 mg GAE (100 g DW)^−1^, and that of freeze-dried samples ranged from 40.1 to 23.7 mg GAE (100 g DW)^−1^. The total flavonoid content (TFC) of hot-air-dried samples ranged from 88.0 to 21.2 mg QE (100 g DW)^−1^, and that of freeze-dried samples ranged from 40.4 to 17.4 mg QE (100 g DW)^−1^, indicating that the antioxidant content of hot-air-dried samples was significantly higher (Figure 3(A1,A2)). The observed increase may be due to heat-induced inactivation of polyphenol oxidase, which reduces the degradation of phenolics, and to moisture loss, which concentrates antioxidant compounds per unit mass [48,49].

Ultrasound-assisted extraction significantly enhanced both TPC and TFC compared to magnetic stirring (*p* < 0.05), likely due to the synergistic effects of cavitation and sponge-like cell disruption. Antioxidant activities are shown in Figure 3(A3–A5). The hot-air-ultrasound group (HA-U) exhibited the strongest antioxidant capacities, with DPPH, FRAP, and ABTS^+^ activities reaching 185.5 and 42.3 mg TE (100 g DW)^−1^, 35.8 and 49.3 mg TE (100 g DW)^−1^, and 10.9 and 12.2 mg TE (100 g DW)^−1^ for PP extract (PPE) and LP extract (LPE), respectively [50]. Notably, the PPEs exhibited stronger DPPH scavenging, while the LPEs outperformed in FRAP and ABTS^+^ assays. This divergence may be attributable to the assay mechanisms—small-molecule phenolic acids are more reactive in DPPH, whereas LPE is rich in glycosylated flavonoids such as hesperidin and eriocitrin [10]. Therefore, in subsequent research, regardless of the extraction source of pineapple peel (100 mesh, pH 4.1 ± 0.1) or lemon peel (100 mesh, pH 3.9 ± 0.1), HA-U was used.

The duration of extraction significantly influenced both yield and the economics of the process. Figure 3B illustrates that the total phenolic content (TPC) and total flavonoid content (TFC) of both PPE and LPE initially increased and subsequently decreased, reaching a maximum at 30 min 49.5 and 23.5 mg GAE (100 g DW)^−1^; 54.7 and 80.2 mg QE (100 g DW)^−1^, representing increases of 102.4% and 32.6%; 63.94% and 133.1%, respectively. Antioxidant activities reached their maximum at 30 min, with DPPH, FRAP, and ABTS^+^ activities recorded as 192.1 and 48.9 mg TE (100 g DW)^−1^, 19.2 and 46.4 mg TE (100 g DW)^−1^, and 11.8 and 14.5 mg TE (100 g DW)^−1^, respectively, indicating an increase. The findings indicate that ultrasound-assisted extraction for 30 min resulted in an increase in antioxidant activities ranging from approximately 14.8% to 260.6% when compared to the 10-min treatment.

The findings indicate that ultrasound-assisted extraction for 30 min resulted in an increase in antioxidant activities ranging from approximately 14.8% to 260.6% when compared to a 10-min treatment. This finding aligns with prior research on pineapple peel extraction using acidified ethanol, which indicated that total polyphenol content reached its peak at 30 min, exhibiting a maximum increase of 27.0% [11]. Prolonged extraction may result in the degradation or structural alteration of phenolics, thereby reducing their antioxidant effects [11,51].

The efficiency of extraction is influenced by particle size, primarily due to its impact on surface area. Figure 3C illustrates that optimal extraction occurred at average particle sizes of 96.6 µm for PPE and 91.7 µm for LPE (*p* < 0.05). At these particle sizes, total phenolic content (TPC) reached 49.5 and 29.2 mg GAE (100 g DW)^−1^, while total flavonoid content (TFC) reached 54.7 and 80.6 mg QE (100 g DW)^−1^. This represents increases of 25.2% and 81.4% in TPC, and increases of 56.04% and 53.67% in TFC, respectively. The DPPH radical-scavenging activity measured 187.4 and 40.0 mg TE (100 g DW)^−1^, while the FRAP activity was recorded at 21.0 and 48.8 mg TE (100 g DW)^−1^. Additionally, the ABTS^+^ activity was 11.6 and 13.0 mg TE (100 g DW)^−1^, respectively. The results indicate that the application of 100 mesh micropowders led to a significant enhancement in antioxidant activity, with improvements ranging from 11.4% to 156.9% when compared to 60 mesh powders. This finding aligns with the results of Chen et al. [12], which indicated that soybean powders with reduced particle sizes demonstrated significantly elevated total flavonoid contents, with the smallest particle group exhibiting a 28.2-fold increase. This supports the notion that smaller particles enhance the solvent–solute contact area, thereby improving extraction yield and reproducibility [12,52].

**Figure 4:**
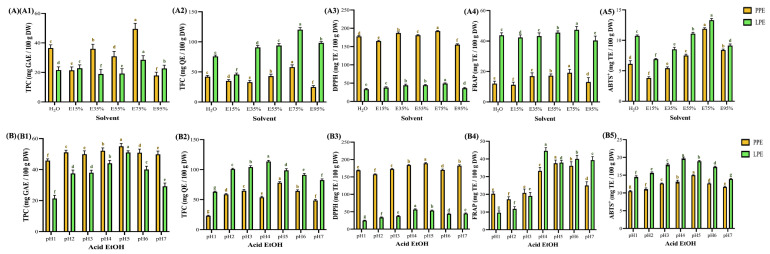
Analysis of functionalities and antioxidant activities of pineapple and lemon peel extracts under different solvents (**A**) and acidified ethanol (**B**). All samples were prepared using 100-mesh sieved peel micropowder and extracted for 30 min with ultrasound-assisted extraction, with acidified ethanol in row B prepared using 75% ethanol. (1) total polyphenols, (2) total flavonoids, (3) 2,2-diphenyl-1-picrylhydrazyl radical scavenging, (4) ferric ion reducing antioxidant power, and (5) 2,2′-azino-bis(3-ethylbenzothiazoline-6-sulfonic acid) radical scavenging. Data are presented as means ± standard deviations. (*n* = 3). Different letters (a–g) indicate statistically significant differences in the same column (*p* < 0.05). Abbreviation description: PPE and LPE denote pineapple and lemon peel extracts, respectively. EtOH and Acid EtOH denote the samples used for ethanol extraction and acidified ethanol extraction, respectively.

Solvent polarity also influences the selectivity of antioxidant components. Ethanol is widely used for its safety and bioactivity, including its radical scavenging, anti-glycation, and antimicrobial properties. As reported in Lai et al. [53], 50–70% ethanol provides optimal DPPH scavenging and anti-glycation activities. Figure 4A shows that increasing ethanol concentration enhanced TPC and TFC in PPE up to a threshold range of 49.5–18.0 and 28.5–18.9 mg GAE (100 g DW)^−1^, respectively, and in LPE up to 58.2–25.4 and 120.2–45.7 mg QE (100 g DW)^−1^, respectively, but further increases to 95% led to a decline (*p* < 0.05). Similar patterns were seen for DPPH, FRAP, and ABTS^+^, with ranges of 192.2–155.3 and 49.0–36.3 mg TE (100 g DW)^−1^, 19.2–11.2 and 47.4–40.4 mg TE (100 g DW)^−1^, and 11.9–3.8 and 13.3–6.9 mg TE (100 g DW)^−1^ for PPE and LPE, respectively, and the highest overall polyphenol and antioxidant yields were obtained at 75% ethanol (*v*/*v*). The findings align with those of Zamper et al. [11], who indicated that the total polyphenol content was greater with a 1:1 ethanol-to-water ratio of 343.04 mg GAE (100 g DW)^−1^ than with single solvents of 219.7–281.9 mg GAE (100 g DW)^−1^, underscoring the influence of solvent polarity on extraction selectivity optimization. Furthermore, water–ethanol mixtures influence cellular permeability and infusion efficiency [54,55]. Acidified ethanol improves membrane permeability and flavonoid stabilization [56]. Figure 4B illustrates the effects of pH on extract quality. The TPC of PPE and LPE ranged 55.0–45.8 and 51.0–21.3 mg GAE (100 g DW)^−1^, and the TFC of PPE and LPE ranged 78.2–23.1 and 113.6–63.1 mg QE (100 g DW)^−1^, respectively. Maximum values were obtained at pH 5 (PPE) and pH 4 (LPE), with corresponding DPPH, FRAP, and ABTS^+^ activities of 189.2 and 56.6 TE (100 g DW)^−1^, 37.6 and 44.6 TE (100 g DW)^−1^, and 15.0 and 19.7 mg TE (100 g DW)^−1^, respectively. These results support previous findings that mildly acidic conditions suppress polyphenol oxidase activity, improve phenolic retention, and enhance thermal stability [12,14,56]. As reported in Ruenroengklin et al. [15], analogous pH-dependent behavior for litchi pericarp was observed, indicating that the highest total phenolic content (TPC) and antioxidant activities occurred at pH 4.0, while lower values were noted at both elevated and reduced pH levels. The absolute values varied due to differences in plant matrix and extraction conditions; however, the observed trend is consistent with the current study, underscoring the significance of optimizing pH to enhance phenolic yield and antioxidant efficacy.

In this study, citric acid was used as a food-grade acidifier to adjust the pH of the 75% ethanol solutions, allowing precise modulation of extraction conditions. Its application not only ensured safe and reproducible acidification but also contributed to the enhanced antioxidant activity observed under mildly acidic conditions.

### 3.4. Principal Component Analysis

**Figure 5:**
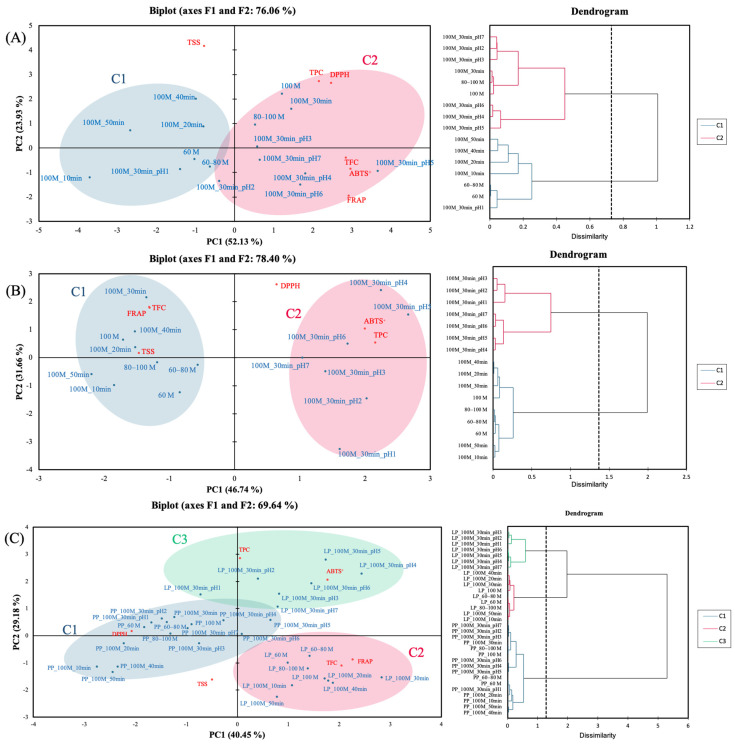
Principal component analysis of antioxidants and their capacities of pineapple peel extracts (**A**), lemon peel extracts (**B**), and both (**C**) within different average particle sizes, extraction times, and pH. 60, 60–80, 80–100, and 100 M denote filtered with different mesh sieves, respectively. 10, 20, 30, and 40 min denote different extraction times, respectively. pH 1, 2, 3, 4, 5, 6, and 7 denote different pH levels of acidified ethanol, respectively. PP, LP denote pineapple peel powder, lemon peel powder, hot-air drying, and freeze-drying, respectively. TPC, TFC, TSS, FRAP, DPPH, and ABTS^+^ denote total polyphenol content, total flavonoid content, total soluble solids, ferric ion reducing power, 2,2-diphenyl-1-picrylhydrazyl, and 2,2′-azino-bis(3-ethylbenzothiazoline-6-sulphonic acid) free radical-scavenging activity, respectively.

To further elucidate the relationships between extraction parameters and the antioxidant as well as physicochemical properties of PPE and LPE, PCA was performed and complemented by agglomerative hierarchical clustering (AHC) for sample classification. Variables included in the PCA model were particle size, extraction time, pH, TSS, TPC, TFC, and antioxidant (DPPH, FRAP, and ABTS^+^) activity (Figure 5). For PPE (Figure 4A), the first two principal components (F1 and F2) explained 52.13% and 23.93% of the total variance, respectively, accounting for a cumulative contribution of 76.06%. AHC grouped the samples into two distinct clusters. Cluster 1 (C1) primarily consisted of samples extracted for 10, 20, 40, and 50 min, with larger particle sizes and pH 1-acidified ethanol, which showed higher TSS levels and were distributed on the left side of the PCA plot. Cluster 2 (C2) included samples extracted for 30 min with smaller particle sizes and pH 2–7-acidified ethanol, characterized by higher TPC, TFC, and antioxidant activities, and located on the right side of the PCA plot. These findings indicate that extraction time and polyphenol yield do not exhibit a strictly linear relationship [57]. Overall, extraction time and pH, particularly within the 80–100 mesh screen, were the most influential variables affecting the antioxidant potential of PPE.

For LPE (Figure 5B), principal components F1 and F2 explained 46.74% and 31.86% of the total variance, respectively, with a cumulative variance of 78.40%. The AHC results divided the samples into two clusters. Cluster 1 included samples extracted with neutral solvents and was characterized by higher TFC, FRAP, and TSS values and was positioned on the left side of the PCA plot. Cluster 2 consisted of acidified ethanol extracts with a 30-min extraction time, showing higher TPC, DPPH, and ABTS^+^ activities, and was located on the right side of the plot. Among these, pH 4 and 5 extracts aligned closely with TPC and DPPH vectors, supporting their superior phenolic extraction efficiency and radical-scavenging performance, consistent with the trends observed in Figure 4B. As in PPE, extraction time and pH were identified as major factors influencing the antioxidant potential of LPE.

When PPE and LPE datasets were combined (Figure 5C), F1 and F2 explained 40.45% and 29.18% of the total variance, respectively, for a cumulative total of 69.64%. AHC differentiated three distinct clusters: Cluster 1 comprised all PPE extracts and was positioned in the lower left quadrant and was characterized by higher DPPH values; Cluster 2 contained pH-unadjusted LPE extracts and was located in the lower right quadrant and marked by higher TFC and FRAP values; and Cluster 3 consisted of acidified ethanol extracts of LPE and was located in the upper quadrant due to elevated TPC and ABTS^+^ levels. These findings suggest that acidified ethanol and extraction time are critical parameters influencing the clustering behavior and antioxidant performance of both peels. Overall, the micrograde size of PP and LP accelerates the release of polyphenols, leading to LPE demonstrating superior antioxidant capacity compared to PPE, suggesting a richer composition of bioactive compounds in LP. The subsequent section explores the specific phenolic and flavonoid components contributing to this functional potential.

### 3.5. Pearson Correlation Analysis

**Figure 6:**
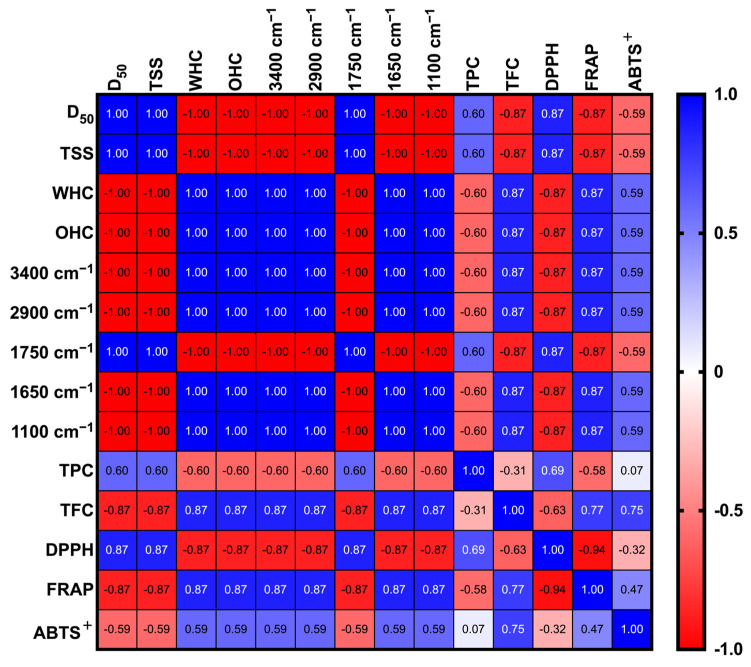
Pearson correlation matrix of physicochemical properties, characteristics, and functionalities between 100 mesh sieved peel powder and 75% acidified ethanol extracts. The pH of acidified ethanol is approximately 4–5. Pearson correlation matrix showing relationships between functional properties and metabolite abundance. Positive and negative associations are color-coded. D_50_, TSS, WHC, and OHC denote average particle size, total soluble solids, water-holding capacity, and oil-holding capacity. TPC, TFC, TSS, FRAP, DPPH, and ABTS^+^ denote total polyphenol content, total flavonoid content, total soluble solids, ferric ion reducing power, 2,2-diphenyl-1-picrylhydrazyl, and 2,2′-azino-bis(3-ethylbenzothiazoline-6-sulphonic acid) free radical-scavenging activity, respectively.

The data of HA-PP and HA-LP samples that passed through the 100-mesh sieve were integrated and subjected to Pearson correlation analysis. In Figure 6, D_50_ exhibited a strong positive correlation with DPPH radical-scavenging activity (r = 0.87) and an equally strong negative correlation with TFC and FRAP reducing power (r = −0.87 for both). These findings indicate that smaller particles facilitate flavonoid extraction and enhance FRAP performance by increasing the solvent–solute contact area, whereas larger particles may still retain phenolic acids that preferentially contribute to DPPH activity [13]. TSS was correlated positively with DPPH activity (r = 0.87) but negatively with FRAP and TFC (both r = −0.87), suggesting that the compounds responsible for TSS differ structurally from those driving flavonoid-associated antioxidant effects, thus highlighting the selective extraction behavior. WHC and OHC showed positive correlations with TFC and FRAP (r = 0.87), implying that these physical properties promote the retention and extraction of flavonoids possessing amphiphilic characteristics and, consequently, enhance physiological functionality.

In the case of FTIR spectral features, the absorbance bands at 3400 cm^−1^ (O–H stretching), 2900 cm^−1^ (C–H stretching), and 1100 cm^−1^ (C–O–C stretching) were positively correlated with TFC and FRAP (r = 0.87), underscoring the close association of these functional groups with flavonoid glycosides or other polyphenolic derivatives. Notably, the bands at 1750 cm^−1^ (ester carbonyl) and 1650 cm^−1^ (O–H bending of bound water) were correlated positively with TPC (r = 0.60), implying that these regions reflect the presence of esterified phenolic acids, such as *p*-coumaric and ferulic acids, in the extracts (Figure 2F; Table 2). TPC was correlated positively with DPPH activity (r = 0.69) but showed a moderate negative correlation with TFC (r = −0.31), indicating that phenolic acids contribute more prominently than flavonoids to DPPH radical quenching, consistent with the mechanistic differences between antioxidant assays (Figure 3A). FRAP and ABTS^+^, both electron-transfer-based assays, were positively intercorrelated (r = 0.47).

Collectively, these correlations highlight the critical roles of particle size, solvent accessibility, and specific chemical functionalities in governing the antioxidant efficacy of PPE and LPE.

### 3.6. Bioactive Composition of Pineapple and Lemon Peel Extracts

**Figure 7:**
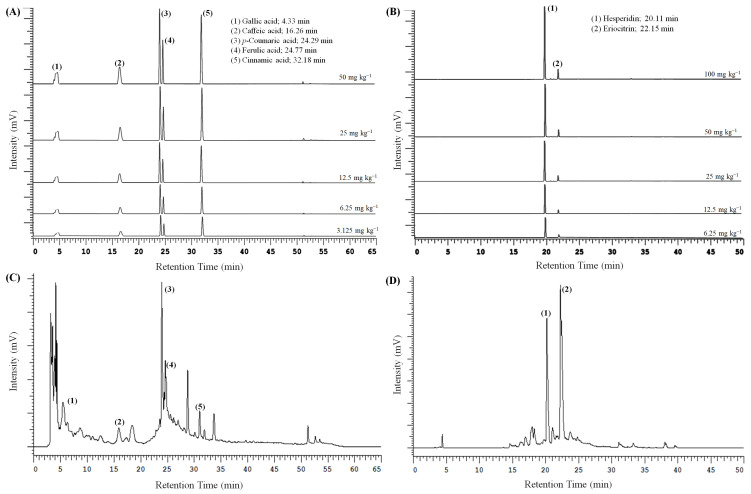
HPLC chromatograms of phenolic acids (**A**) and flavonoids (**B**) standards and phenolic acid in pineapple peel extracts (**C**) and polyphenol in lemon peel extracts (**D**). The samples used in (**C**) were prepared using 100 mesh peel powder and extracted for 30 min using ultrasound-assisted extraction with 75% acidified ethanol (pH 5), and those used in (**D**) were prepared using 100 mesh peel powder and extracted for 30 min using ultrasound-assisted extraction with 75% acidified ethanol (pH 4).

Based on the PCA results, the optimal extraction conditions for PPE and LPE were identified as PP_100 M_30 min_pH5 and LP_100 M_30 min_pH4, respectively, and were further analyzed using HPLC-UV. As shown in Figure 7 and Table 2, the extract from PP_100 M_30 min_pH5 contained gallic acid (9.6 µg/mg DW), caffeic acid (7.0 µg/mg DW), *p*-coumaric acid (4.7 µg/mg DW), ferulic acid (10.4 µg/mg DW), and cinnamic acid (0.7 µg/mg DW) (Table 3). These compounds are known for their antioxidant, anti-inflammatory, antiallergic, antithrombotic, antimicrobial, cardioprotective, anticancer, and antidiabetic properties [58,59,60]. Compared to a previous study of ultrasound-assisted extraction of PE with acidified ethanol for 30 min, the present study achieved higher extraction efficiency, likely due to the optimized pH condition [10].

In the case of LP_100 M_30 min_pH4, the hesperidin and eriocitrin content was 65.7 µg/mg DW and 23.2 µg/mg DW, respectively, both of which exhibit strong antioxidant potential [14,61]. Notably, the hesperidin content obtained in this study exceeded that of a previous study using pressurized hot water extraction (10.34 MPa, 160 °C, 5 min), suggesting that mild, environmentally friendly extraction conditions can outperform thermally intensive methods in preserving flavonoid integrity [62].

**Table 3:** Bioactive compounds with demonstrated antioxidant activity in the referenced studies.

Compound	Source	Demonstrated Antioxidant Activity	Study
Cinnamic acid	Cinnamon, other plants	Scavenges free radicals and reactive oxygen species; contributes to oxidative stress reduction.	[63]
Gallic acid	Berries, tea, other plants	Strong antioxidant activity; increases total antioxidant capacity; inhibits lipid peroxidation.	[64]
Caffeic acid	Coffee, fruits, vegetables	Reduces oxidative stress in brain tissue, inhibits ROS accumulation, protects neurons from Aβ-induced damage.	[65]
*p*-Coumaric acid	Fruits, vegetables, cereals	Decreases lipid peroxidation, increases antioxidant enzyme activity (SOD, CAT), reduces oxidative stress in the liver and plasma.	[66]
Ferulic acid	Whole grains (wheat, rice bran), corn	Strong antioxidant; inhibits lipid peroxidation; stabilizes cell membranes; protects from UV-induced oxidative stress.	[67]
Hesperidin	Citrus fruits (oranges, lemons)	Reduces inflammation; scavenges ROS; protects tissues	[68]
Eriocitrin	Lemon peel, citrus fruits	Protects mitochondria; reduces oxidative stress	[69]

### 3.7. Contour Plot Visualization

**Figure 8:**
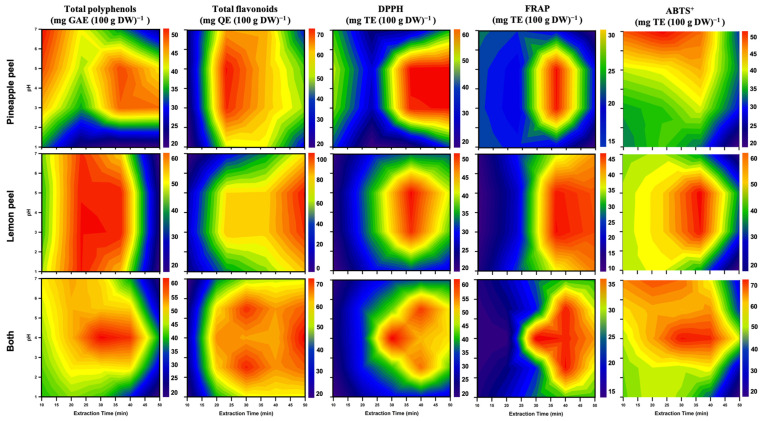
Contour plots of antioxidants and their capacities within different pH solvents and extraction time on pineapple peel, lemon peel, and both extracts. All peel micropowder was filtered through a 100-mesh sieve.

To comprehensively assess the impact of pH and extraction time on the antioxidant activities, including TPC, TFC, and radical-scavenging capacities (DPPH, FRAP, ABTS^+^), of PPE and LPE, two-dimensional contour plots were generated (Figure 8). These visualizations serve as a practical tool for optimizing functional compound extraction from agricultural by-products in food industry applications.

The first row of Figure 8 depicts the contour distribution for PPE. TPC and TFC reached their relative maxima within the pH range of 4–6 and extraction times of 20–35 min. DPPH and FRAP values peaked prominently around pH 5 and 30–40 min, indicating this range as the optimal antioxidant extraction window. This trend is likely attributable to the enhanced stability of polyphenolic compounds and suppression of polyphenol oxidase activity under mildly acidic conditions [55]. Acidic extractions can inhibit polyphenol oxidase and peroxidase activity, thereby preventing the oxidative degradation of phenolic compounds and enhancing the measurable TPC as well as antioxidant capacity, such as DPPH radical-scavenging activity. Thus, we considered phenolic acids to exhibit improved solubility and chemical stability under low pH conditions, which may further contribute to the enhanced extraction efficiency observed [58,59].

The second row of Figure 8 shows pronounced TPC and TFC peaks for LPE at pH 3–6 and within extraction times of 25–40 min. DPPH and ABTS^+^ activities also reached maxima in the pH 5–6 range, suggesting efficient flavonoid release under these conditions. As noted by a previous study [69], LP is particularly rich in flavonoids such as hesperidin and eriocitrin, which show high stability and antioxidant potential in mildly acidic environments.

The third row of Figure 8 presents an integrated visualization of both PPE and LPE responses, highlighting the synergistic influence of pH and extraction time on the yield and activity of bioactive compounds. The optimal conditions, as identified from the heat maps, were extractions conducted at room temperature for 30–40 min using citric acid–adjusted acidified ethanol at pH 5–6. The compositional differences of by-products from various varieties can be minimized using micronization technology. Additionally, contour plots indicate that optimal antioxidant characterization is achieved by extracting ethanol with citric acid at pH 5–6 for 30–40 min. This outcome serves as a significant reference for industrial extraction. These data-driven visualizations not only provide a practical basis for extraction optimization but also align with the goals of Industry 4.0 by enabling intelligent, digitally managed production of functional ingredients. Combined with PCA, this multi-parameter modeling strategy offers a valuable foundation for future integration with machine learning systems to support data-informed antioxidant extraction decisions.

### 3.8. Study Limitations

Although acidified ethanol is generally regarded as safe (GRAS), residual solvent levels were not quantified in the present work and should be addressed in future scale-up studies. Antioxidant capacities were evaluated exclusively through in vitro assays (DPPH, FRAP, ABTS^+^); thus, bioavailability and in vivo efficacy remain to be verified. Future studies should further integrate in vivo antioxidant activities and response surface methodology to support environmental-friendly extraction optimized strategy for various food by-products.

## 4. Conclusions

Ultrasound-assisted extraction with citric acid acidified ethanol provided an environmental-friendly, high-yield route to recover polyphenols from pineapple and lemon peels. Under lower micrograde powder (91.7–96.6 µm), extraction pH and time dominated performance, with 75% ethanol adjusted by citric acid to pH 5–6 and 30–40 min giving maximal yields. Under these conditions, lemon peel outperformed pineapple peel owing to its richer hesperidin and eriocitrin content, translating to superior DPPH, FRAP, and ABTS^+^ activities. Contour plots and PCA pinpointed the synergistic parameter space, demonstrating the value of data-driven optimization for scalable, intelligent processing. Overall, the use of citric acid as a safe acidifier, combined with ultrasound and micronization, confirms the potential of peel as a functional material for applications in the food industry, offering a valuable and sustainable use for agricultural by-products that aligns with the goals of environmental sustainability and waste reduction.

## Figures and Tables

**Table 1:** Extraction conditions for micropowder from pineapple and lemon peels.

Variables	Conditions
Ultrasound parameter	40 kHz, 500 ± 50 W
Extraction time	10, 20, 30, 40, 50 min
Particle size	>60, 60–80, 80–100, <100 mesh
Ethanol ratio in deionized water	0, 15, 35, 55, 75, 95%
Acidified ethanol ^†^	pH 1, 2, 3, 4, 5, 6, 7

^†^ Acidified ethanol (75%, *v*/*v*) was obtained by dissolving citric acid directly into the solvent. Citric acid concentrations of 0.005–2.000 g/5 mL (0.1–40%; *w*/*v*) generated solutions with measured pH values of 6, 5, 4, 3, 2, and 1, respectively. pH was monitored with a calibrated glass electrode; values served as operational indicators for comparing extraction conditions (Figure S1).

**Table 2:** Bioactive composition of pineapple and lemon peel extracts.

Sample	Phenolic Acids (µg (mg DW)^−1^)					Flavonoids (µg mg (DW)^−1^)	
	Gallic Acid MW 170.12 (g mol^−1^)	Caffeic Acid MW 180.16 (g mol^−1^)	*p*-Coumaric Acid MW 164.05 (g mol^−1^)	Ferulic Acid MW 194.18 (g mol^−1^)	Cinnamic Acid MW 148.15 (g mol^−1^)	Hesperidin MW 610.56 (g mol^−1^)	Eriocitrin MW 596.53 (g mol^−1^)
PP_100 M_ 30 min_pH5	9.164 ± 0.021	6.988 ± 0.031	4.733 ± 0.025	10.355 ± 0.049	0.663 ± 0.022	ND	ND
LP_100 M_ 30 min_pH4	ND	ND	ND	ND	ND	65.685 ± 0.018	23.195 ± 0.037

Data are presented as means ± standard deviations (*n* = 3). PP_100 M_30 min_pH5 and LP_100 M_30 min_pH4 denote the acidic ethanol extracts (pH 5) of pineapple peel powder (<100 mesh) and acidic ethanol extracts (pH 4) of lemon peel powder (<100 mesh) under 30-min extraction, respectively. MW and ND denote molecular weight and not detectable, respectively.

## Data Availability

The original contributions presented in the study are included in the article; further inquiries can be directed to the corresponding author.

## Supplementary Materials

The following supporting information can be downloaded at: https://www.mdpi.com/article/10.3390/foods14162872/s1; Figure S1. Preparation ratios of acidified ethanol. The solvent used was a 75% aqueous ethanol solution; Figure S2. The calibration curves of gallic acid (A), ferulic acid (B) *p*-coumaric acid (C), cinnamic acid (D), caffeic acid (E); Figure S3. The calibration curves of hesperidin (A) and eriocitrin (B); Figure S4. Particle size distribution and span values of 100 mesh sieved pineapple and lemon peel micropowders.
