# Supplementary material for: Micronization Combined Ultrasound-Assisted Extraction Enhances the Sustainability of Polyphenols from Pineapple and Lemon Peels Utilizing Acidified Ethanol
Source: Foods. 2025 Aug 19;14(16):2872. doi: 10.3390/foods14162872 (PMC12385742; doi:10.3390/foods14162872)
Supplement: Supplementary file 1 [file foods-14-02872-s001.zip › foods-3769185-supplementary.pdf]

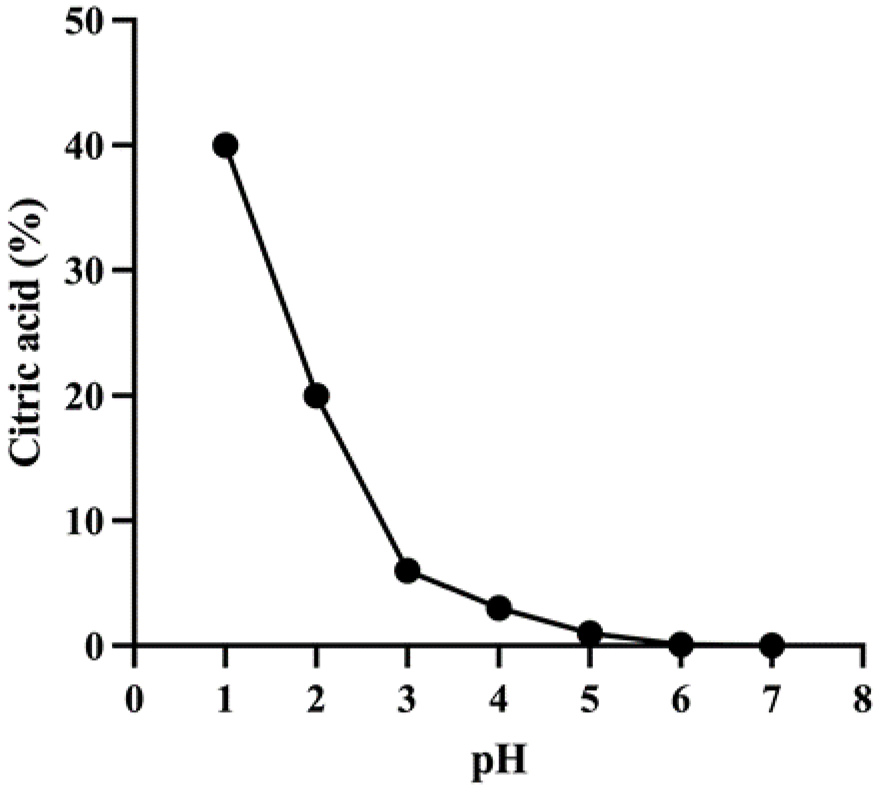

Figure S1. Preparation ratios of acidified ethanol. The solvent used was a 75% aqueous ethanol solution.

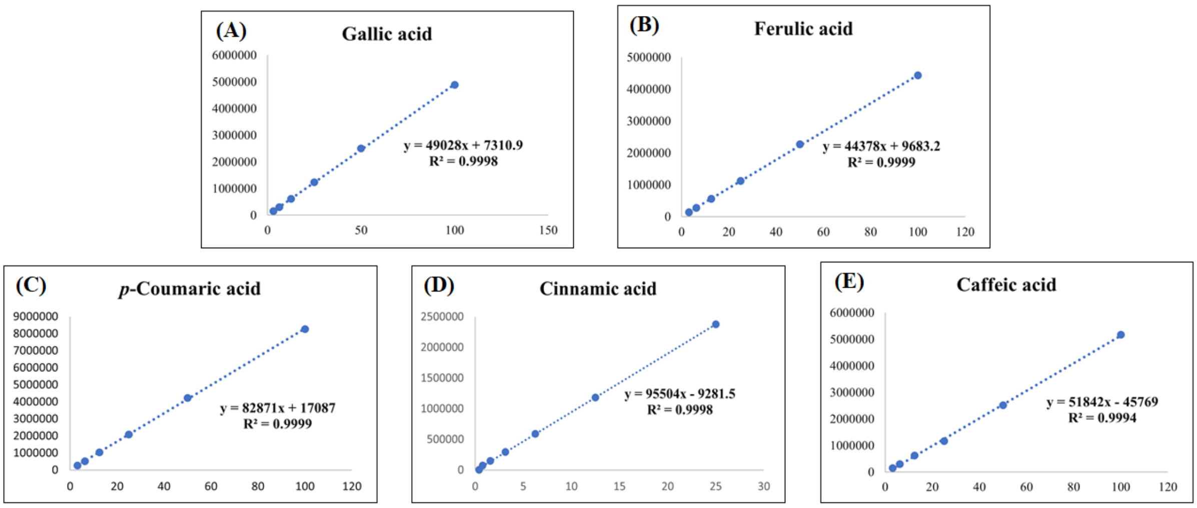

Figure S2. The calibration curves of gallic acid (A), ferulic acid (B). *p*-coumaric acid (C), cinnamic acid (D), caffeic acid (E). The calibration curves were constructed using concentrations ranging from 3.125 to 100.000 μg (mg DW)$^{-1}$ for gallic acid, caffeic acid, p-coumaric acid, and ferulic acid, 0.391 to 25.000 μg (mg DW)$^{-1}$ for cinnamic acid.

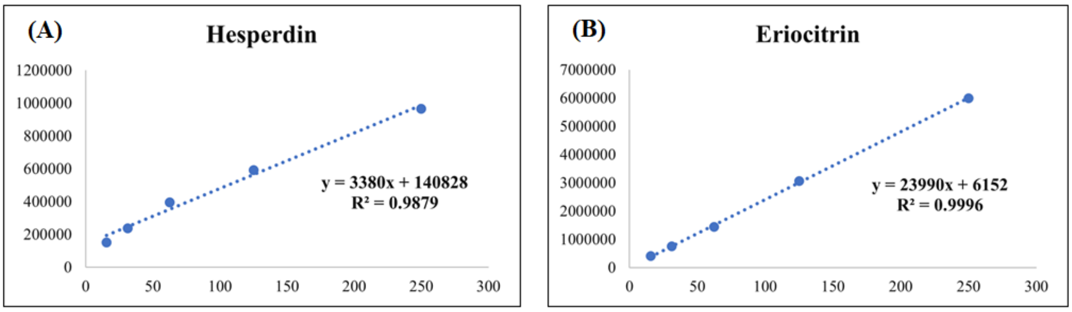

Figure S3. The calibration curves of hesperidin (A) and eriocitrin (B). The calibration curves were constructed using concentrations ranging from 3.125 to 100.000 μg (mg DW) $^{-1}$ for hesperidin and eriocitrin.

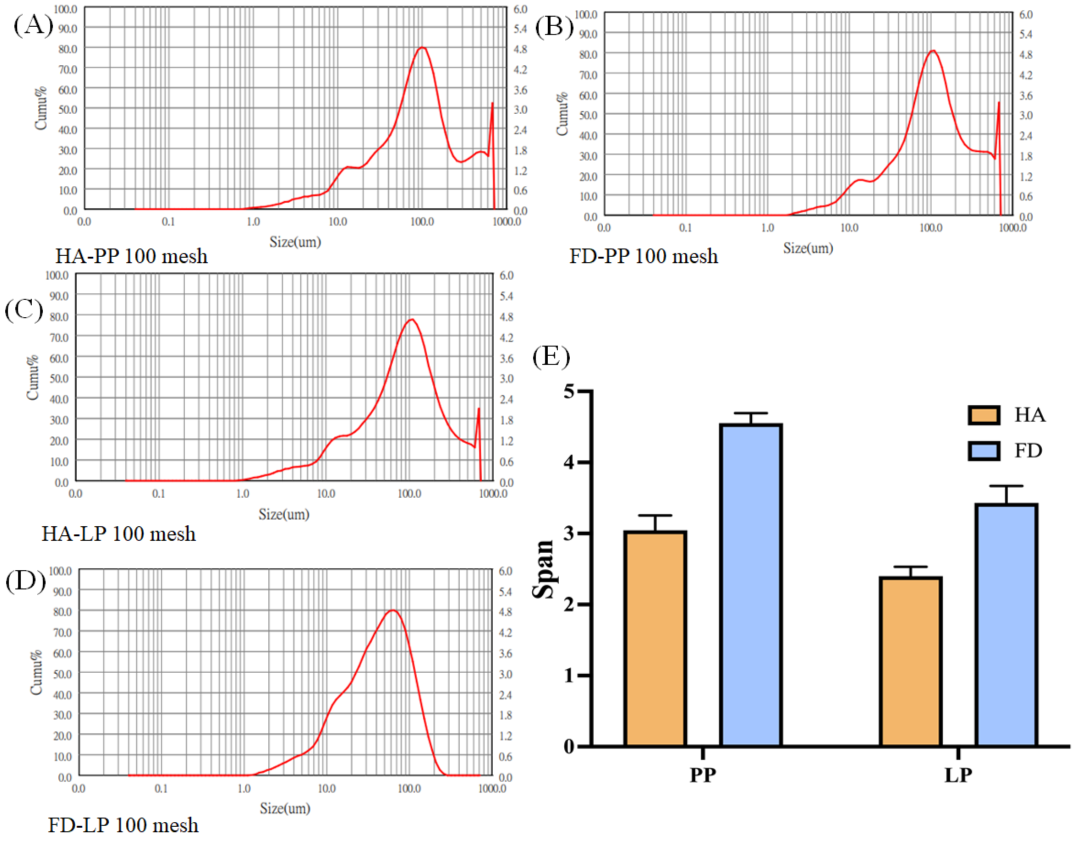

Figure S4. Particle size distribution and span values of 100 mesh sieved pineapple and lemon peel micropowders. HA, FD, PP, and LP denotes to hot air drying, freeze drying, pineapple peel powder, and lemon peel powder.
